# A Case of Suspected Scombroid Poisoning in Intensive Care Staff: Is It Anaphylaxis?

**DOI:** 10.7759/cureus.116634

**Published:** 2026-09-21

**Authors:** William Hamilton, Helen Ivatt, Gwendolen Cartwright

**Affiliations:** 1 Intensive Care Unit, Morriston Hospital, Swansea, GBR; 2 Intensive Care Unit, Glangwili General Hospital, Carmarthen, GBR

**Keywords:** adrenaline-refractory shock, allergy and anaphylaxis, histamine fish poisoning, non-ige-mediated food allergy, scombroid fish poisoning

## Abstract

Scombroid poisoning (histamine fish poisoning) is an uncommon foodborne illness that frequently mimics immunoglobulin E-mediated anaphylaxis. We report a case of a previously well 28-year-old intensive care nurse who developed abrupt facial flushing, wheeze, dizziness, and hypotension after consuming canned tuna. Initial observations demonstrated sinus tachycardia and hypotension. She was treated according to established anaphylaxis guidelines with two doses of intramuscular adrenaline, intravenous crystalloid resuscitation, and subsequent adrenaline infusion in intensive care. She recovered rapidly and was discharged the following day. Serial serum mast cell tryptase measurements at approximately one hour, six hours, and 24 hours after symptom onset were all within normal limits. In the absence of previous allergy history and given the temporal association with tuna ingestion, scombroid poisoning was considered as a possible diagnosis. This case highlights the difficulty of distinguishing severe histamine fish poisoning from anaphylaxis in the acute setting and reinforces that life-threatening presentations should initially be managed according to anaphylaxis protocols until an alternative diagnosis can be established.

## Introduction

Scombroid poisoning, also known as histamine fish poisoning, is caused by ingestion of fish containing excessive concentrations of histamine produced through bacterial decarboxylation of histidine during inadequate storage [1]. Tuna, mackerel, and bonito are among the species most frequently implicated. Clinical manifestations occur rapidly after ingestion and commonly include facial flushing, urticaria, headache, gastrointestinal disturbance, bronchospasm, and hypotension. As these features closely resemble immunoglobulin E (IgE)-mediated anaphylaxis, differentiation at presentation is often impossible. Although most cases are mild and self-limiting, severe cardiovascular collapse has been described [2]. Distinguishing scombroid poisoning from anaphylaxis is important for both initial and ongoing management. However, emergency treatment should never be delayed while attempting to distinguish the two conditions.

## Case presentation

A 28-year-old female intensive care nurse became acutely unwell during a work shift shortly after eating a tuna salad prepared using canned tuna. This individual was the only person to consume any of this tuna. Her medical history included well-controlled asthma treated with salbutamol as required. Approximately 30 minutes after lunch, she developed intense facial flushing associated with wheeze, dizziness, and urticaria. Assuming she was experiencing an asthma exacerbation, she self-administered salbutamol via a metered-dose inhaler. Despite these measures, her symptoms rapidly progressed, and within five minutes of symptom onset, she was having trouble standing unassisted. Colleagues assisted her onto a bed where initial observations demonstrated sinus tachycardia of 128 beats·min⁻¹ and hypotension of 76/43 mmHg. An emergency call was activated because of concern regarding anaphylactic shock [3].

She was immediately positioned supine with leg elevation, intravenous access was established, and 1 litre of intravenous crystalloid was administered. Two sequential doses of intramuscular adrenaline (500 micrograms) were required because of persistent hypotension. She was also given a 4 mg chlorphenamine tablet prior to adrenaline administration. She was transferred to the emergency department resuscitation area before admission to intensive care for ongoing management with an adrenaline infusion. Her haemodynamic status improved rapidly following vasopressor therapy. The adrenaline infusion was discontinued after several hours without recurrence of hypotension or respiratory symptoms. No recurrence of symptoms occurred, and she was discharged home the following day. Electrocardiography demonstrated sinus tachycardia only (Figure 1).

**Figure 1:**
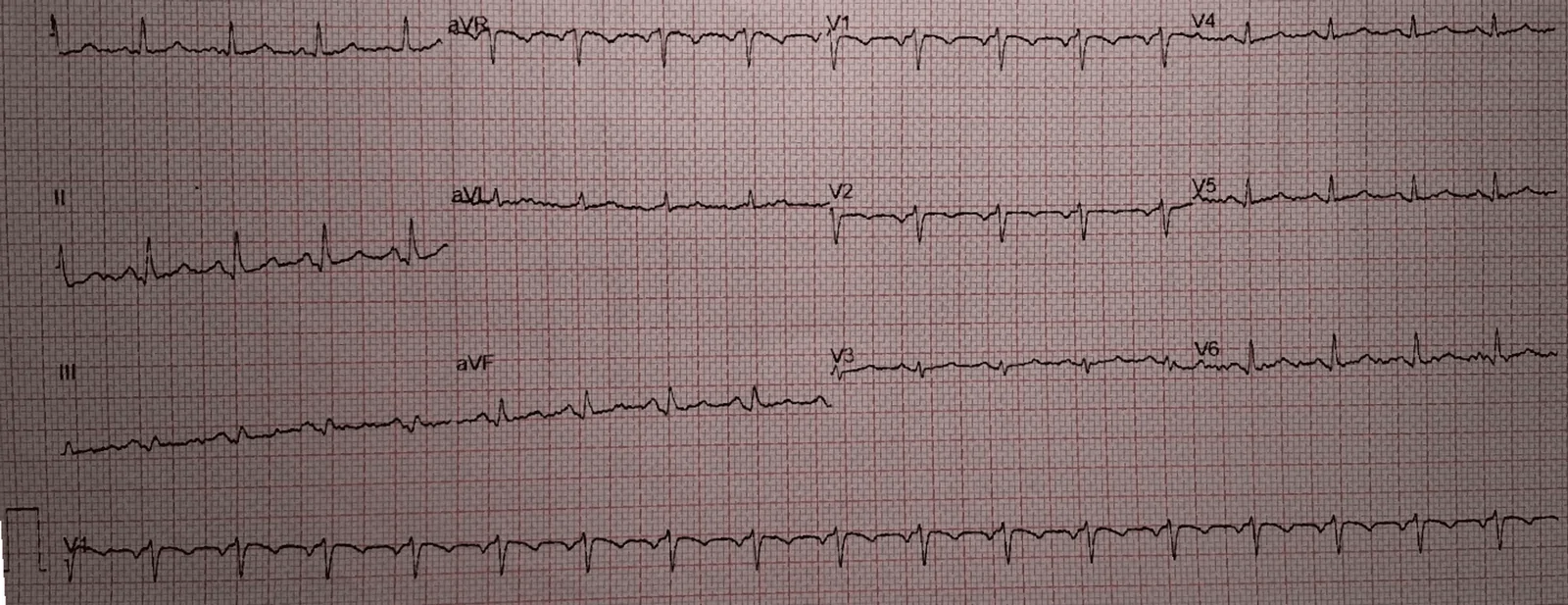
ECG showing sinus tachycardia.

Serum mast cell tryptase concentrations were obtained according to local anaphylaxis investigation protocols at one hour, six hours, and 24 hours after symptom onset. All serum measurements were within the normal reference range (Table 1).

**Table 1:** Patient's serum tryptase levels measured at one hour, six hours, and 24 hours after symptom onset.

Parameters	Patient values	Reference range
Tryptase at 1 hour	4 mcg/L	<15 mcg/L
Tryptase at 6 hours	3 mcg/L	<15 mcg/L
Tryptase at 24 hours	3 mcg/L	<15 mcg/L

The close temporal relationship between canned tuna ingestion and symptom onset, together with persistently normal serial tryptase concentrations, prompted reconsideration of the diagnosis following clinical recovery [2]. The initial differential diagnosis included IgE-mediated food-induced anaphylaxis, scombroid (histamine fish) poisoning, acute asthma, vasovagal/syncope, septic or other distributive shock, and idiopathic anaphylaxis. The combination of bronchospasm, hypotension, and cardiovascular collapse fulfilled accepted clinical criteria for anaphylaxis, making immediate treatment mandatory [4].

Following recovery, several features favoured suspected scombroid poisoning: onset within minutes of consuming canned tuna, prominent facial flushing, absence of previous allergy history, persistently normal serial serum tryptase concentrations, and rapid complete recovery.

The patient recovered completely. The adrenaline infusion was successfully discontinued within several hours. She remained haemodynamically stable overnight without recurrence of symptoms and was discharged home the following day. Following multidisciplinary review of the clinical course and investigation results, suspected scombroid poisoning was considered more likely than IgE-mediated anaphylaxis.

## Discussion

Scombroid poisoning results from ingestion of preformed histamine generated during bacterial spoilage of fish that has not been maintained at appropriate storage temperatures. Unlike IgE-mediated allergy, histamine toxicity affects any individual consuming the contaminated food and therefore does not require previous sensitisation. The clinical syndrome typically develops within minutes to one hour following ingestion and commonly presents with flushing, headache, palpitations, urticaria, and gastrointestinal symptoms. Bronchospasm and hypotension may produce a clinical picture that is indistinguishable from severe anaphylaxis [5].

This case demonstrates the potential severity of histamine fish poisoning. Despite being a young, previously healthy healthcare professional, the patient developed distributive shock requiring repeated intramuscular adrenaline and vasopressor infusion in intensive care. Such severe cardiovascular compromise has been reported only rarely. An additional learning point concerns interpretation of serum tryptase. Persistently normal serial tryptase concentrations supported an alternative diagnosis but did not exclude anaphylaxis. Food-related anaphylaxis frequently occurs without significant elevations in serum tryptase because mast cell activation may be relatively localised or involve pathways that produce minimal systemic release [6]. Consequently, clinicians should avoid using a normal tryptase result to retrospectively dismiss an episode that fulfilled clinical diagnostic criteria.

To further their recovery, the patient was referred via their general practitioner to a specialist allergy service for formal assessment of possible fish or seafood allergy. This evaluation included a detailed allergy history together with consideration of skin-prick testing and serum-specific IgE testing to relevant fish and seafood allergens. At the time of writing, the results from these investigations were unavailable. Such investigations were considered important given the severity of the presentation and the diagnostic overlap between scombroid poisoning and IgE-mediated anaphylaxis.

Serum histamine concentrations were not measured because this is not part of the standard diagnostic work-up available within this institution and requires specialised sample collection and processing with limited availability. In addition, no analysis of the implicated fish was possible because the meal had been completely consumed before symptom onset. Measurement of histamine concentrations within the fish, confirmation of bacterial spoilage, or direct testing of the implicated food source may have provided stronger evidence in support of scombroid poisoning, while correlation with subsequent allergy testing may have further helped to exclude IgE-mediated fish allergy. Consequently, the final diagnosis was based on the characteristic clinical presentation, temporal relationship to fish ingestion, normal serial tryptase concentrations, and the results of subsequent specialist allergy assessment.

Recognition of suspected scombroid poisoning remains important after the acute event because the implications differ considerably from those of IgE-mediated allergy. Patients with histamine fish poisoning generally do not require lifelong avoidance of fish and should not automatically receive a diagnosis of seafood allergy. Appropriate counselling may therefore prevent unnecessary dietary restriction and inappropriate prescription of emergency medication. Nevertheless, emergency management should remain unchanged. Patients presenting with respiratory compromise and hypotension after fish ingestion should be treated immediately as having anaphylaxis until an alternative diagnosis has been established. Early administration of adrenaline remains the cornerstone of management and should never be delayed while attempting to distinguish between these conditions.

## Conclusions

This case highlights the diagnostic challenge of distinguishing suspected scombroid poisoning from IgE-mediated anaphylaxis. The patient's rapid onset of flushing, bronchospasm, and hypotensive shock following tuna ingestion necessitated immediate treatment according to established anaphylaxis guidelines, including adrenaline administration and intensive care support. Clinicians should remain aware that histamine fish poisoning can mimic life-threatening anaphylaxis, but emergency management should prioritise prompt treatment of anaphylaxis until an alternative diagnosis can be confidently established.
